# *Eugenia calycina* and *Eugenia stigmatosa* as Promising Sources of Antioxidant Phenolic Compounds

**DOI:** 10.3390/plants13152039

**Published:** 2024-07-24

**Authors:** Juliana Dara Rabêlo Silva, Henrique Silvano Arruda, Amanda Cristina Andrade, Patrícia Berilli, Felipe Tecchio Borsoi, Yaneth Machaca Monroy, Marili Villa Nova Rodrigues, Klicia Araujo Sampaio, Glaucia Maria Pastore, Mario Roberto Marostica Junior

**Affiliations:** 1 Department of Food Science and Nutrition (DECAN), Faculty of Food Engineering (FEA), University of Campinas (UNICAMP), Campinas 13083-862, São Paulo, Brazil; hsilvanoarruda@gmail.com (H.S.A.); andradenut@gmail.com (A.C.A.); pbberilli@gmail.com (P.B.); felipe.tecchio@gmail.com (F.T.B.); glaupast@unicamp.br (G.M.P.); 2 Department of Food Engineering and Technology (DETA), School of Food Engineering (FEA), University of Campinas (UNICAMP), Campinas 13083-862, São Paulo, Brazil; yaneth@unicamp.br (Y.M.M.); klicias@unicamp.br (K.A.S.); 3 Pluridisciplinary Center for Chemical, Biological and Agricultural Research (CPQBA), University of Campinas (UNICAMP), Paulínia 13148-218, São Paulo, Brazil; marilivn@unicamp.br

**Keywords:** *Eugenia* fruits, *Myrtaceae*, polyphenols, anthocyanins, berries

## Abstract

In this study, *Eugenia calycina* and *Eugenia stigmatosa*, native Brazilian berries, were explored regarding their proximal composition, bioactive compounds, and antioxidant activities. The edible parts of both fruits presented a low content of lipids, proteins, and carbohydrates, resulting in a low caloric value (<70 kcal/100 g fw). *E. stigmatosa* fruit showed a high total fiber content (3.26 g/100 g fw), qualifying it as a source of dietary fiber. The sugar profile was mainly monosaccharides (glucose, fructose, and rhamnose). Significant contents of total phenolics and flavonoids, monomeric anthocyanins and, condensed tannins, were observed in both fruits. *E. calycina* contains a high level of anthocyanins, primarily cyanidin-3-glucoside (242.97 µg/g). Other phenolic compounds were also found, the main ones being rutin and ellagic acid. In contrast, *E. stigmatosa* is mainly composed of rutin and gallic acid. Furthermore, these fruits showed expressive antioxidant activity, evidenced by ORAC, FRAP, and ABTS. These *Eugenia* fruits are promising sources of bioactive compounds and have a low caloric and high dietary fiber content, making them interesting options for inclusion in a balanced diet, contributing to the promotion of health and the valorization and conservation of Brazilian biodiversity.

## 1. Introduction

Brazil has a wide biodiversity of plant species, with more than 46,000 species spread across different biomes (Amazon rainforest, Pantanal, Cerrado, Caatinga, Pampa, and the Atlantic Forest). This biodiversity corresponds to one of the country’s main sources of resources [1]. Among the various native Brazilian fruits, *Eugenia calycina*, also known as “cereja-do-cerrado,” “cerejinha,” and “cereja-do-campo,” is an attractive fruit with an intense purple color and sweet taste. Another notable native Brazilian fruit is *Eugenia stigmatosa* known as “guarimi” and “guamirim vermelho.” This berry has a red or intense orange color and a sweet flavor accompanied by a certain astringency. Both belong to the *Myrtaceae* family. *E. stigmatosa*, endemic to Brazil, is primarily concentrated in the Atlantic Forest region, with occurrences predominantly in the southeastern states (Rio de Janeiro, São Paulo), as well as confirmed occurrences in Bahia, Paraná, and Santa Catarina. *E. calycina*, non-endemic, is distributed across a wide area of Brazil, predominating in the phytogeographic domains of the Cerrado, Atlantic Forest, and Pampa. It occurs in a variety of vegetation types, from forests to high-altitude fields and rocky outcrop vegetation [2].

Fruits are widely recognized as essential sources of nutrients, performing a crucial role in promoting health and well-being and significantly contributing to the prevention of various chronic diseases such as cardiovascular diseases, type 2 diabetes, obesity, cancer, and others. Scientific studies consistently support the benefits of a diet rich in fruits, vegetables, and legumes in reducing the risk of several adverse conditions, including severe cardiovascular diseases and both cardiovascular and non-cardiovascular mortality [3]. A prospective cohort study conducted by Miller et al. [3] confirms these findings, demonstrating an association between increased consumption of these foods and a significant decrease in the risk of total mortality.

The genus *Eugenia*, the largest in number of species among neotropical *Myrtaceae*, comprises approximately 1050 species distributed from southern Mexico to Uruguay and Argentina, with a small number also found in Africa, Southeast Asia, and Pacific islands. In Brazil, *Eugenia* represents the genus with the highest plant diversity, totaling 407 species. Among these, 256 are found in the Atlantic Forest, the primary phytogeographic domain where the genus is most diverse. The Amazon and Cerrado biomes also host significant numbers, with 108 and 83 species, respectively [2].

The main *Eugenia* species studied are: *E. uniflora* L., *E. stipitata* Mc Vaughn, *E. brasiliensis* Lam., *E. pyriformis* Cambess and *Eugenia dysenterica* DC. These *Eugenia* fruit species are renowned for their potential high content of bioactive compounds, which may include phenolic compounds such as tannins and proanthocyanidins, as well as phenolic acids like gallic acid, ellagic acid, and chlorogenic acid. Additionally, they may contain flavonoids such as myricetin, quercetin, rutin, and catechin; anthocyanins like cyanidin, delphinidin, malvidin, and pelargonidin; and carotenoids such as lycopene, lutein, α-carotene, and β-carotene, among others [4]. It is important to note that the presence and concentration of these compounds can vary among different fruits, each possessing a unique chemical composition.

These compounds present a range of biological activities, including antioxidant and anti-inflammatory effects that are associated with the prevention of various diseases, such as neurocognitive disorders, cardiovascular diseases, cancer, and diabetes [5,6,7,8,9,10]. Therefore, encouraging the greater inclusion of these fruits in the diet is a recommended and effective strategy for promoting health and improving well-being.

Extraction is the starting point for studies involving bioactive compounds. The conventional method of extracting compounds from a solid matrix is solid–liquid extraction, using solvents such as methanol, ethanol, ethyl acetate, and acetone [11]. Recently, a new concept called green extraction has gained preference. This concept promotes sustainable processes, such as reducing energy consumption and using alternative, less environmentally harmful solvents. Additionally, it allows for the combination of techniques and solvents in different processes [11].

The choice of extraction method is crucial in the recovery and purification of phenolic compounds from plant matrices, and it should be based on simplicity, versatility, cost, and the ability to preserve the compounds [12]. Emerging extraction methods are increasingly being used instead of conventional methods. These methods are preferred due to their rapid extraction rate, high recovery yield, preservation of thermosensitive compounds, lower processing temperatures, and the use of safe organic solvents [11].

Among the emerging methods, ultrasound-assisted extraction stands out for its simplicity and efficiency. This method reduces solvent and energy consumption compared to conventional techniques, and it is also fast and reproducible. It is valued for being economical and environmentally friendly, offering high reproducibility in a short period, whereas other traditional techniques require longer times to complete the process [11].

Implementation of the initiatives aimed at improving quality of life and promoting sustainability is intrinsically linked to achieving the Sustainable Development Goals, thereby contributing to promoting population well-being [13]. Additionally, these efforts contribute to advancing scientific knowledge, guiding future research on health implications and facilitating the development of novel strategies for disease prevention and health promotion. Furthermore, the goals encourage environmental sustainability and the appreciation of Brazilian biodiversity.

This study aimed to analyze for the first time the chemical composition of two native Brazilian fruits (*E. calycina* and *E. stigmatosa*), focusing on their proximate composition, sugar, and phenolic compound profile and content, and antioxidant activity. It is expected to emphasize the nutritional value of these fruits to promote their consumption, thereby encouraging a healthier lifestyle and contributing to sustainable development efforts.

## 2. Results and Discussion

### 2.1. Proximate Composition of Eugenia Fruits

For the first time, results are presented on the proximate composition of the edible parts of *E. calycina* and *E. stigmatosa*. Both species exhibited a high moisture content, especially *E. calycina*, which contributes to the greater juiciness of this fruit. It was observed that higher ash content was found in *E. stigmatosa*, indicating a higher concentration of minerals in this fruit. As expected, these species do not contain significant amounts of lipids or proteins, comprising less than 1% of their weight, while carbohydrates are the most represented macronutrients in these fruits. These values contribute to a low caloric value (~49–62 Kcal/100 g), making them attractive from a nutritional point of view. Moreover, knowing the macronutrient content of foods can help health professionals make decisions when developing a dietary plan. The proximate composition of *E. calycina* and *E. stigmatosa* is presented in Table 1.

Regarding dietary fiber, a significant predominance of the insoluble fraction over the soluble was observed in both fruits studied. Insoluble fibers are efficient for the proper functioning of the digestive system, as they increase fecal volume due to reduced intestinal transit time and help with the elimination of waste. Soluble fibers promote slow gastric emptying and thus delay the absorption rate, reducing glucose absorption and serum cholesterol levels [14].

Daily dietary fiber intake has been linked to human health and well-being, preventing several diseases such as obesity, diabetes, cardiovascular disorders, and gastrointestinal tract issues. According to the World Health Organization (WHO), the daily recommendation for fiber intake is 25 g to achieve its functional benefits is necessary [15]. Therefore, consumption of 100 g of *E. calycina* should contribute 6.64% of the daily recommendation, while *E. stigmatosa* would contribute 13.04%. According to the Food and Drug Administration (FDA), a food that provides 10% to 20% of the recommended daily intake (RDI) of a nutrient per serving is classified as a “good source” [16]. In this context, *E. stigmatosa* can be considered and classified as a good source of dietary fiber.

In a preclinical study, a research group investigated the prebiotic and antidiabetic potential of jaboticaba peel (*Plinia cauliflora* (Mart.) Kausel), a native Brazilian berry fruit rich in dietary fiber and polyphenols. Jaboticaba peel integrated into a healthy diet promoted a recognized prebiotic action with modulation of the profile of gut bacteria, which was associated with an improvement in glucose metabolism [17].

The findings of that study present results very similar to those reported for other *Eugenia* species. *E. uniflora* L. showed moisture content of 81.2–84.7 g, ash of 1.1–2.4 g, proteins of 1.1–1.4 g, lipids of 0.4–0.5 g, and carbohydrates of 12.9–14.8 g/100 g fw [18]. Similarly, *E. brasiliensis* [19], presented moisture content of 83.0–90.2 g, ash content of 0.2–0.6 g, proteins of 0.3–0.7 g, lipids of 0.01–0.2 g, carbohydrates of 4.2–7.6 g, fibers of 1.0–4.2 g, and caloric value of 33.1–45.6 kcal/100 g fw. Furthermore, *Eugenia* Sp1 [20] had a moisture content of 84.9 g, ash of 0.6 g, proteins of 1.0 g, lipids of 2.7 g, carbohydrates of 10.7 g, and energy of 71.8 kcal/100 g fw. These findings are consistent with the present study’s results for Brazilian *Eugenia* berries.

### 2.2. Sugar and Oligosaccharide Content in Eugenia Fruits

The identification and quantification of sugars and oligosaccharides present in *E. calycina* and *E. stigmatosa* using HPAEC-PAD detected the presence of monosaccharides (fructose, glucose, and rhamnose) in both *Eugenia* fruits (Table 2).

The glucose:fructose ratio was approximately 1.02:1.00, being slightly higher in *E. calycina*. The absorption of fructose is maximized when the glucose:fructose ratio is equal to or greater than 1:1, making it more tolerable due to its complete absorption. Therefore, the levels of fructose that will or will not be absorbed depend on the total amount of fructose ingested and the substances consumed concomitantly [21].

Thus, knowing the amount of fructose present in excess relative to glucose in food is essential for predicting the potential for malabsorption [22]. In some individuals, the delivery of fructose to the small intestine and its subsequent rapid fermentation by colonic bacteria can exacerbate intestinal discomfort, due to the production of gases (H_2_, CO_2_, and CH_4_). These gases can trigger symptoms such as abdominal distension, discomfort or abdominal pain, and altered bowel habits, associated with irritable bowel syndrome (IBS) and other intestinal disorders [23]. In this context, individuals, especially patients with IBS, with or without fructose malabsorption, may need to make dietary choices that significantly reduce these intestinal symptoms.

Quantitative variations in individual and total sugar content between fruits can be attributed to different stages of ripeness, since throughout ripening, fructose, glucose, and total sugar increase continuously, while sucrose gradually decreases [24]. Nehring et al. [25] evaluated *E. brasiliensis* from different cultivation sites and maturation stages, observing variations between 38.1 to 52.8 mg/g for fructose and 29.9 to 41.7 mg/g for glucose, with no sucrose detected in the samples, corroborating the findings for the profile and sugar content in the native Brazilian species of *Eugenia*.

Furthermore, external factors such as agricultural practices and climatic variations between cultivation locations can also affect the composition of nutrients and other compounds in the fruit [26].

Concerning disaccharides, *Eugenia* fruits do not present significant quantities, with only maltose detected in *E. stigmatosa* in quantities less than 1 mg/g fw. Trace amounts of maltooligosaccharides (maltotriose) were identified in *E. stigmatosa*, and fructooligosaccharides (GF2 and GF3) were found in *E. calycina*. However, these values were below the detection limits of the method, making quantification impossible

### 2.3. Phytochemical Content and Antioxidant Activity in Eugenia Fruits

Bioactive compounds, such as polyphenols are secondary metabolites in plants known for their remarkable antioxidant capacity. These compounds primarily act by donating electrons or hydrogen atoms to intermediate radicals. In addition to their antioxidant properties, they are also known for their anti-inflammatory [27] and antineoplastic effects and can improve biochemical and metabolic markers associated with health benefits [8,9,10].

Both *Eugenia* species exhibited significant amounts of total phenolics and flavonoids. Notably, *E. calycina* displayed higher content of anthocyanins, with significantly higher levels (*p* < 0.01). Conversely, *E. stigmatosa* contained significantly higher levels of condensed tannins (*p* < 0.01). The results for bioactive compounds and antioxidant activity assays are shown in Table 3.

On antioxidant activity assays, no statistical difference was found between the FRAP assay values (*p* <0.05). However, *E. stigmatosa* showed higher antioxidant activity than *E. calycina* in both ABTS and ORAC assays (*p* < 0.01). Interestingly, in the ORAC, *E. calycina* exhibited almost double the antioxidant activity of *E. stigmatosa*, which may be attributed to its high anthocyanins content.

No previous studies have reported antioxidant analysis for *E. stigmatosa*. However, a similar *Eugenia* species, *E. punicifolia*, has been studied by Ramos et al. [28]. In their study, red ripe fruits demonstrated a total phenolic content of 36.6 mg GAE/g of extract (lyophilized). Converting the result of the present study to dry weight (35.33 mg GAE/g) shows a similarity with the value reported by the authors. However, the antioxidant activity assessed by the ABTS method was lower (174.00 µmol TE/g of extract lyophilized) compared to the findings of this study when converted to dry weight (308.74 µmol TE/g). Conversely, Braga et al. [29] reported lower phenolic levels (1.46 mg GAE/g fw).

Araujo et al. [30,31] investigated the phenolic content of *E. calycina* and identified lower phenolic values than those found in this study (5.82–18.14 mg in dry weight and 2.04 mg/fw). Additionally, they noted that the phenolic content and antioxidant capacity in *E. calycina* varied according to the extraction method and time used [30].

The presence and quantity of antioxidant compounds in plant material can be influenced by various environmental factors. These include species differences, geographic locations, maturation stages, soil characteristics, extraction techniques, solvent types, extraction duration, temperature variations, and other elements [32,33].

Bonin et al. [34] and Nehring et al. [25] studied the ripening stage of *E. brasiliensis* and demonstrated that the composition of nutrients and bioactive compounds can be influenced by the ripening stage of the fruit. The authors found a substantial increase in sugar content and a significant increase in bioactive compounds. Particularly for anthocyanins, the content was higher when the fruit was fully ripe, with anthocyanin values at medium- and fully ripe stages ranging from 135.4 to 518.6 mg/100 g of fresh weight [25]. Besides maturation stage, extraction conditions are crucial factors that can significantly influence the total content of bioactive compounds. Thus, the use of ultrasound for extractions can impact the process of obtaining bioactive compounds, where operational conditions of the equipment such as extraction time, temperature, and power, among others, can have a substantial impact on the extraction of anthocyanins and other compounds, and may generate different effects in various matrices [35].

Pinela et al. [36] compared the efficacy of ultrasound-assisted extraction (UAE) with heat-assisted extraction (HAE) in obtaining anthocyanins from the calyx of *Hibiscus sabdariffa*. They observed that the unconventional method UAE resulted in significantly higher yields, with a recovery of approximately three times more anthocyanins compared to HAE (51.76 vs. 20.86 mg/g of extract).

On the other hand, a recent study by Albuquerque et. al. [37] showed that HAE was more efficient for peels of fruits from *E. brasiliensis* and *E. involucrata* compared to UAE. The results indicated that while UAE produced extracts with higher yield (*E. brasiliensis* UAE 38% and *E. involucrata* 33% vs. HAE 38% and 31%, respectively), extraction by heat resulted in extracts with higher anthocyanin content in a shorter processing time (*E. brasiliensis* HAE 268.0 vs. UAE 229.0 mg/g of extract and *E. involucrata* HAE 20.0 vs. UAE 16.8, respectively).

Extraction conditions such as techniques used, types of solvents employed, and duration of the process can selectively alter the composition of bioactive compounds in the extract, necessitating a more in-depth study to select optimal conditions based on the compounds of interest for investigation.

Pearson’s correlation was used to determine covariance between phytochemical content and antioxidant activities (Table 4).

A negative correlation was found for total phenolics and tannins with ORAC (r = −0.99 and −0.97, respectively) and a positive correlation for anthocyanins and flavonoids with ORAC (r = 0.99 and 0.98, respectively). This indicates that the greater anthocyanin content, the higher the antioxidant activity measured by ORAC, suggesting that these compounds are mainly responsible for the increased antioxidant activity in *E. calycina*. The high antioxidant capacity of anthocyanins is due to their unique structure, which allows efficient donation of hydrogen atoms from the phenolic hydroxyl group (OH) and facilitates electron donation. The electronic stabilization provided by the π–electron conjugation in flavonoids results in an excellent ability of anthocyanins to neutralize free radicals [38]. This gives anthocyanins remarkable antioxidant activity and is also associated with other beneficial activities, such as the prevention of cardiovascular diseases, antitumor effects, anti-inflammatory properties, and various other health benefits [39].

The presented results clearly show that the primary mechanism of action for both extracts is more efficient via hydrogen atom transfer, since the principle of the ORAC_FL_ assay involves terminating the free radical chain reaction by inhibiting hydrogen transfer. Thus, this method exhibits the antioxidant capacity by blocking the chain reaction process of free radicals [40]. Another factor to consider in this method is that the free radicals used in the experiment are similar to those produced by the body, effectively approximating a biological system compared to other assays, such as ABTS, which also primarily acts by electron transfer, although it can slow hydrogen atom transfer, or FRAP, which performs simple electron transfer, measuring the ability of the bioactive substance to reduce Fe^3+^ to Fe^2+^, without direct biological correlation of antioxidant activity [40]. Thus, it is evident that both fruits in this study exhibit better antioxidant capacity when the method employed involves hydrogen atom donation, as there was no correlation with the FRAP assay.

Furthermore, another point to highlight in this study is that ORAC_FL_ measures the capacity of a compound to inhibit peroxyl radicals. These radicals are highly reactive species that can damage cellular components such as DNA, proteins, and polyunsaturated fatty acids present in cell membranes. Thus, peroxyl radicals are involved in triggering and/or worsening various comorbidities, including cancer development, inflammatory processes, and cardiovascular diseases, among others [41].

The results this study, provide insights into the primary mechanism of action of the antioxidant capacity of both *Eugenia* species. These findings can be further investigated at a biological level in in vivo models for a better understanding of the biological mechanisms and more realistic effects on their in vivo impacts.

In part, corroborating this study, Toshima et al. [42] analyzed different berries (raspberry, blackberry, and wild Japanese *Rubus* spp.) and found that the antioxidant capacity measured by different assays (FRAP, ORAC, and DPPH) had high positive correlations with both the total polyphenol and the anthocyanin contents. However, it is necessary to consider the limitations of these methods, as they do not show specificity for a compound or a specific class, so other compounds not analyzed may interfere with the total antioxidant capacity and influence the result.

In this study, the *Eugenia* species studied have proven to be promising sources of phenolic compounds, with a significant antioxidant capacity. These findings offer perspectives for future investigations with these fruits, starting with in vitro studies, such as cell cultures, which can provide more specific responses regarding the action of these compounds on cells like macrophages and neoplastic cells, potentially elucidating their anti-inflammatory, antioxidant, and antitumor effects, for example. This opens the door to investigating the effects and possible mechanisms of action involved in different cell lines. Subsequently, this information could guide more robust investigations using animal models, such as rats and mice, commonly used for this type of study, allowing for a better understanding of the mechanisms of action and effects of *Eugenia* spp. at the biological level and in various conditions, such as obesity, inflammatory bowel diseases, cancer, diabetes, and more.

### 2.4. Phenolic Compound Profile and Content in Eugenia Fruits

The quantitative analysis by HPLC-DAD revealed that the phenolic compound rutin was predominant in both fruits, being approximately five times more abundant in *E. calycina* (*p* < 0.05). Rutin is a flavonoid with recognized antioxidant and anti-inflammatory properties. It has been well elucidated that flavonoids can contribute to the prevention of neurodegenerative diseases, cardiovascular diseases, and cancer, among others [43]. The results are shown in Table 5.

The second major compound in *E. calycina* was ellagic acid, which was not detected in *E. stigmatosa*. Ellagic acid is a non-flavonoid polyphenol naturally found in certain fruits, such as berries. These fruits can provide ellagic acid in the form of ellagitannins, which make up approximately 60% (in red raspberries) of the total phenolic compounds present. There is evidence that ellagic acid can reduce symptoms of chronic metabolic diseases, including dyslipidemia, insulin resistance, type 2 diabetes, and non-alcoholic fatty liver disease [44]. Araujo et al. [30] found that *E. calycina* pulp predominantly contained ellagic acid (73.40%), myricitrin (13.24%), and quercetin-3-O-galactoside (7.15%).

On the other hand, gallic acid was observed in significant proportions in *E. stigmatosa*, but not in *E. calycina*. Quercetin was approximately five times more abundant in *E. calycina* (*p* < 0.05). Additionally, compounds exclusively identified in *E. calycina* included protocatechuic acid, gentisic acid, and caffeic acid, whereas quercetin, trans-cinnamic acid, and kaempferol were found exclusively in *E. stigmatosa*. The total phenolic content based on the standards used was higher in *E. calycina*.

Ramos et al. [28] found higher values of gallic acid in *E. punicifolia* (45–160 µg/g) than those reported in this study for the *Eugenia* fruits analyzed. The authors also verified the presence of quercetin-3-O-rhamnoside and kaempferol-7-O-rhamnoside in addition to linoleic acid, β-glucogallic acid, and myricetin-3′-rhamnoside. Braga et al. [29] found gallic acid to be predominant (95%) among the phenolic acids present in the fruit of *E. punicifolia*, a higher proportion than that found in this study, while the other acids (protocatechuic acid, syringic acid, p-coumaric acid, and ferulic acid) represented only 2% of the fruit.

Regarding anthocyanins, cyanidin-3-O-β-D-glucoside was predominant, representing 97% of the total anthocyanins identified chromatographically in *E. calycina*, while *E. stigmatosa* showed a significantly lower content. This is the first report in the scientific literature showing the anthocyanin composition of these fruits. In other *Eugenia* spp., such as *E. brasiliensis* [45,46] and *E. jambolana* Lam [47], cyanidin-3-O-β-D-glucoside was the main anthocyanin present, and other anthocyanins such as the delphinidin aglycone were also identified. Additionally, pelargonidin-3-O-β-D-glucoside was identified in *E. involucrata* [39].

The antioxidant profile of *E. brasiliensis* was investigated in detail by Teixeira et al. [48]. Five anthocyanins were identified (cyanidin aglycone, cyanidin-3-xyloside/arabinoside, cyanidin-3-O-β-D-glucoside, cyanidin-3-galactoside, and delphinidin hexoside). In this study, the major anthocyanin was similar to *E. calycina*, with cyanidin-3-O-β-D-glucoside responsible for 82–89% of total anthocyanins.

Recently, a published meta-analysis found that purified anthocyanins at doses ≥ 320 mg/day can modulate the serum levels of markers related to inflammation, reducing the levels of CRP, TNF-α, and IL-6 [49]. Another similar study showed that regular consumption of foods containing anthocyanins can improve vascular function, lipid profile, and antioxidant and anti-inflammatory effects [50].

Although there is no specific recommendation from the Food and Agriculture Organization (FAO) for the daily intake of phenolic substances and their subclasses, the WHO recommends a daily consumption of around 400 g of fruits and vegetables for adults [51]. These foods are naturally rich in bioactive compounds. Therefore, encouraging increased consumption of these foods can strategically contribute to an increased intake of polyphenols in the diet. Though research in this area is extensive, further studies and deeper investigation are needed to better understand the mechanisms involved in their action, especially in clinical models.

*E. calycina* showed significantly higher contents of glucose and rhamnose compared to *E. stigmatosa* (*p* < 0.05), while the fructose content did not differ between the fruits (*p* > 0.05). Fructose is a monosaccharide with low absorption when ingested alone. However, its absorption can be significantly enhanced in the presence of glucose.

## 3. Materials and Methods

### 3.1. Fruit Collection and Botanical Identification

The ripe fruits, which easily detached from the peduncle, were selected based on the intensity of their coloration: intense reddish orange for *E. stigmatosa* and deep purple for *E. calycina*. These were collected in October and November 2022, respectively, in the vicinity of the University of Campinas (*E. calycina*: 22°49′09.0″ S and 47°04′08.0″ W, and *E. stigmatosa*: 22°49′08.0″ S and 47°04′14.0″ W). The use of these fruits was registered in SisGen/Brazil under the protocol number A380866. Botanical identification was carried out by a specialist on the *Myrtaceae* family, and the exsiccates were deposited in the UEC herbarium (Institute of Biology of the State University of Campinas), registered as 212,426 for *Eugenia calycina* Cambess and 212,247 for *Eugenia stigmatosa* DC. The edible parts (peel and pulp) were manually separated from the seed, homogenized with a vertical mixer (Black Decker, M250, Uberaba, Brazil), and stored at −20 °C until analysis.

### 3.2. Ultrasound-Assisted Extraction

The fruit extracts were prepared as previously described by Paludo et al. [52] with minor modifications. In summary, 2 g of homogenized fresh sample was weighed and extracted with acidified hydroethanolic solution (ethanol:water:acetic acid, 60:39.5:0.5, *v*/*v*/*v*) at a solid:liquid ratio of 1:5. The mixture was vortexed for one minute and then subjected to ultrasound-assisted extraction in an ultrasonic bath (Unique, USC—1800A, 40 kHz and 132 W, São Paulo, Brazil) at 30 °C for 30 min. After extraction, the samples were centrifuged (10,732× *g*, 4 °C, 10 min) and stored at −20 °C until analysis.

### 3.3. Proximate Composition

The proximate composition of the fruits was determined by moisture content using oven drying at 105 °C [53], lipid content [54], and ash and protein content according to the American Association of Cereal Chemists (AACC) [55], with a nitrogen conversion factor of 6.25. Total carbohydrates were estimated by calculating the difference between the sum of the previously described components. The total caloric value was calculated using the Merril and Watt equation [56]. The soluble, insoluble, and total dietary fibers were determined by the enzymatic–gravimetric method using a commercial kit (Megazyme©, Bray, Ireland) following the manufacturer’s protocol recommendations.

### 3.4. Chromatographic Analysis of Sugars and Oligosaccharides

The samples were extracted with ultrapure water (1:20, *w*/*v*) using an ultrasound bath (UNIQUE, model UCS-2850, 25 kHz, 120 W, Campinas, SP, Brazil) for 10 min at room temperature. After centrifugation (4000× *g*, 25 min, 5 °C), the supernatants were filtered through 0.22 μm-filter membrane units. The analysis of sugars and oligosaccharides was performed by a high-performance anion exchange chromatography coupled with pulsed amperometric detection (HPAEC-PAD) system model DIONEX ICS-5000 (Thermo Fisher Scientific, Waltham, MA, USA), according to Pereira et al. [57] with some modifications. Sugars (xylitol, mannitol, sorbitol, arabinose, rhamnose, glucose, fructose, and sucrose) were separated on a CarboPac PA1 column (250 × 4 mm i.d., 10 μm particle size, Thermo Fisher Scientific, Waltham, MA, USA) using an isocratic mobile phase (0.12 mol/L NaOH). Fructooligosaccharides and maltooligosaccharides were separated on a CarboPac PA100 column (250 × 4 mm i.d., 8.5 μm particle size, Thermo Fisher Scientific, Waltham, MA, USA) using three mobile phases: 0.2 mol/L NaOH (eluent A), ultrapure water (eluent B), and 0.5 mol/L sodium acetate containing 0.2 mol/L NaOH (eluent C). The elution gradient was performed as follows: 0–2 min, 47% A, 50% B, and 3% C; 2–18 min, 47–10% A, 50% B, and 3–40% C; 18–23 min, 100% C; 23–28 min, 47% A, 50% B, and 3% C. In both analyses, the flow rate was 1 mL/min, the column temperature was kept at 30 °C, and the injection volume was 25 μL. Sugars and oligosaccharides were identified in the samples by comparing the retention times of the standards and the samples. Calibration curves were constructed with commercial standards (0.25–12.50 μg/mL) to quantify the sugars and oligosaccharides in the samples. The content of individual compounds was expressed as grams per 100 g of fresh fruit (g/100 g fw).

### 3.5. Determination of Total Phenolic Content

Total phenolic content was quantified using the Folin–Ciocâlteu colorimetric method [58] with modifications adapted from Vasco et al. [59]. The diluted extract (30 μL) was combined with 10% *v*/*v* Folin–Ciocâlteu reagent (150 μL) and 5% *w*/*v* sodium carbonate (120 μL). The reaction was conducted at 45 °C, with an absorbance reading at 760 nm against a blank in a microplate reader (Biotek Synergy™ HT, Winooski, VT, USA). Gallic acid was used to construct the standard curve, and the results were expressed as milligrams of gallic acid equivalents per gram of fresh fruit (mg GAE/g fw).

### 3.6. Determination of Total Flavonoid Content

Total flavonoid content was determined using the aluminum chloride colorimetric assay [60]. In summary, 100 μL of diluted extract was mixed with 500 μL of ultrapure water, and 30 μL of 5% (*w*/*v*) NaNO_2_ was added to each microtube. After 5 min, 60 μL of 10% (*w*/*v*) AlCl_3_ was added and allowed to stand for 6 min. Finally, 200 μL of 1 M NaOH and 310 μL of ultrapure water were added and mixed. The absorbance was measured at 510 nm against a blank in a microplate reader (Biotek Synergy™ HT, Winooski, VT, USA). Catechin was used to plot the standard curve, and the results were expressed as milligrams of catechin equivalents per gram of fresh fruit (mg CE/g fw).

### 3.7. Total Monomeric Anthocyanins

Total monomeric anthocyanins were determined by the pH-differential method [61]. The extracts were appropriately diluted using potassium chloride buffer (pH 1.0) to present absorbance in the range of 0.4–0.8 and sodium acetate buffer (pH 4.5) in the same proportions. Absorbance was measured in a microplate reader (Biotek Synergy™ HT, Winooski, VT, USA) at 520 and 700 nm. The final absorbance was obtained by Equation (1):

A = [(A520 nm–A700 nm) pH 1.0] − [(A520 nm–A700 nm) pH 4.5](1)

The anthocyanin content was calculated as cyanidin-3-O-glucoside (C3G) equivalents using Equation (2):

(2) $$C(mgC3G/g)=\;\frac{A\times \mathrm{MW}\times \mathrm{DF}}{ɛ}\times L$$

where A = absorbance, C = concentration, MW = molecular weight (449.2), DF = dilution factor, ε = molar absorptivity (26.900 mol L^−1^), and L = path length (cm).

### 3.8. Condensed Tannin Content

Condensed tannin content was determined according to the method described by Arruda et al. [62]. Briefly, 30 μL of diluted extract, 900 μL of 4% (*w*/*v*) vanillin prepared in methanol, and 450 μL of concentrated HCl were mixed and incubated at room temperature for 20 min. The absorbance was measured at 500 nm against a blank in a microplate reader (Biotek Synergy™ HT, Winooski, VT, USA). Catechin was used for the standard curve, and the results were expressed as milligrams of catechin equivalent per gram of fresh fruit (mg CE/g fw).

### 3.9. Antioxidant Activity Assays

#### 3.9.1. Ferric Reducing Antioxidant Power (FRAP)

The FRAP assay was conducted and adapted to the microplate according to Guerra-Ramírez et al. [63]. FRAP solution was prepared by adding 0.3 M acetate buffer at pH 3.6, 10 mM TPTZ, and 20 mM ferric chloride (10:1:1, *v*/*v*/*v*). In a transparent 96-well microplate, an aliquot of 20 µL of the sample, 180 µL of FRAP solution, and 60 µL of deionized water were pipetted, incubated for 30 min at 37 °C and absorbance at 595 nm read against a blank in a microplate reader (Biotek Synergy™ HT, Winooski, VT, USA). The results were expressed as micromoles of Trolox equivalents per gram of fresh fruit (μmol TE/g fw).

#### 3.9.2. Trolox Equivalent Antioxidant Capacity (TEAC) Assay

The TEAC assay is based on the ability of antioxidants in the sample to reduce the ABTS^•+^ radical cation through the transfer of electron or hydrogen atoms. The antioxidant capacity of *Eugenia* fruit extracts was determined based on the method described by Re et al. [64] and modified by Arruda et al. [62]. In summary, the radical cation ABTS^•+^ was prepared by mixing 5 mL of 7 mM ABTS solution and 88 μL of a 145 mM potassium persulfate solution and was kept at rest for 16 h. Subsequently, the ABTS^•+^ radical cation was diluted with ultrapure water until reaching an absorbance of 0.70 ± 0.02 at 734 nm. The extracts of the native Brazilian fruit *Eugenia* (50 μL) were mixed with 250 μL of ABTS^•+^ solution, and the reaction was left to rest at room temperature for 6 min. Absorbance was measured at 734 nm against a blank in a microplate reader (Biotek Synergy™ HT, Winooski, VT, USA). Results were expressed as micromoles of Trolox equivalents per gram of fresh fruit (μmol TE/g fw).

#### 3.9.3. Oxygen Radical Absorbance Capacity (ORAC_FL_) Assay

The ORAC_FL_ test was performed using a method described by Dávalos et al. [65]. In a dark 96-well microplate, 20 μL of diluted extract, blank or Trolox, 120 μL of fluorescein (0.378 μg/mL, pH 7.4), and 60 μL AAPH (108 mg/mL) were mixed. Fluorescence was determined and recorded every minute for 80 min at 37 °C with a microplate reader (Biotek Synergy™ HT, Winooski, VT, USA). The fluorescence filters for excitation and emission wavelengths were 485 and 520 nm, respectively. Results were expressed as micromoles of Trolox equivalents per gram of fresh fruit (μmol TE/g fw).

#### 3.9.4. Chromatographic Analysis of Phenolic Compounds

The bioactive flavonoids and phenolic acids from extracts were analyzed by high-performance liquid chromatography coupled with a photodiode array detector (HPLC-DAD) system (Dionex UltiMate 3000, Thermo Fisher Scientific, Waltham, MA, USA). Separation was performed on an AcclaimTM 120 A C18 column (250 × 4.6 mm i.d., 5 μm particle size, Thermo Fisher Scientific, Waltham, MA, USA) with a gradient program at a 0.5 mL/min flow rate. The column temperature was maintained at 32 °C and the injection volume was 20 μL. The mobile phases consisted of 0.1% formic acid in deionized water (eluent A) and HPLC-grade acetonitrile (eluent B). The gradient was performed as follows: 0–5 min, 5% B; 5–27 min, 5–29% B; 27–33 min, 35% B; 33–45 min, 35–50% B; 45–50 min, 95% B; and 50–60 min, 5% B. Chromatograms were recorded simultaneously at 260, 280, 320, and 360 nm. Identification of individual flavonoids and phenolic acids was based on retention times and spectral characteristics relative to standard compounds. Calibration curves were constructed with commercial standards (0.10–10.00 μg/mL) to quantify the flavonoids and phenolic acids in the samples. The content of individual phenolic compounds was expressed as micrograms per gram of fresh fruit (µg/g fw).

#### 3.9.5. Chromatographic Analysis of Anthocyanins

The anthocyanins in the extracts were analyzed using high-performance liquid chromatography coupled with a photodiode array detector (HPLC-DAD) system (Shimadzu Corporation, Kyoto, Japan). The system consisted of an LC-10AT pump, an SIL-20A HT autosampler, an SPD-M10A VP diode array detector, a DGU-2A degasser, and an SCL-10A VP interface. Data acquisition was carried out using Class VP 5 software. Separation was performed on XBridge Phenyl column (100 × 2.1 mm i.d., 5 μm particle size, Waters Ltd., Milford, MA, USA) with a gradient program at a 0.3 mL/min flow rate. Column temperature was maintained at 30 °C and the injection volume was 10 μL. The mobile phases consisted of 2% formic acid in deionized water (eluent A) and 2% formic acid in HPLC-grade acetonitrile (eluent B). The gradient was performed as follows: 0–10 min, 2% to 8% B; 10–15 min, 8% to 20% B; 15–25 min, 20% to 100% B and maintaining this concentration for 30 min. Chromatograms were recorded at 520 nm. Identification of individual anthocyanins was based on retention times and spectral characteristics relative to standard compounds. Calibration curves were constructed using commercial standards (1.00–50.00 μg/mL) to quantify the anthocyanins in the samples. The content of individual anthocyanins was expressed as micrograms per gram of fresh fruit (µg/g fw).

## 4. Conclusions

This study provides innovative insights into the nutritional and phytochemical characteristics of *E. calycina* and *E. stigmatosa* fruits. For the first time, a detailed analysis of the proximal composition, sugar profile, phenolic compounds, and antioxidant activity of these species was conducted. Both species have low caloric value and are sources of dietary fiber, with *E. stigmatosa* standing out due to its higher fiber content, contributing significantly to the recommended daily intake. The sugar profile revealed that both fruits contain similar levels of fructose and glucose, with *E. calycina* showing a glucose/fructose ratio of 1:1, making it a good option for people with discomfort or poor digestion related to fructose. The analyses revealed distinct profiles of bioactive compounds between the two species. *E. calycina* had higher concentrations of anthocyanins, confirmed by the high concentration of cyanidin-3-glucoside, while *E. stigmatosa* showed higher levels of condensed tannins. The correlation between phenolic compounds and antioxidant capacity indicates that anthocyanins are responsible for the higher antioxidant activity observed in *E. calycina*. The diversity of phenolic compounds, such as rutin in *E. calycina* and gallic acid in *E. stigmatosa*, reflects the different bioactive profiles of these fruits and highlights their various functional properties. The identified compounds have the potential to provide protection against oxidative stress and contribute to the prevention of chronic diseases. These findings offer a solid foundation for future research on the mechanisms of action of these compounds and encourage continued investigation to explore more deeply the benefits of *Eugenia* fruits and their possible therapeutic applications. Additionally, the identification and quantification of bioactive compounds associated with health benefits not only add value to foods but also encourage increased consumption of these fresh foods. This contributes to strengthening and promoting healthy eating habits, encouraging the inclusion of native fruits in the diet and promoting a traditional, healthy, and sustainable diet. This study also reinforces the importance of valuing biodiversity and encourages sustainability in scientific research.

## Figures and Tables

**Table 1:** Proximate composition of edible parts from *Eugenia* fruits.

Parameters	*Eugenia calycina*	*Eugenia stigmatosa*	*p*-Value
Moisture (g/100 g fw)	87.57 ± 0.29	84.73 ± 0.16	<0.001 *
Ashes (g/100 g fw)	0.34 ± 0.02	0.44 ± 0.05	0.039 **
Lipids (g/100 g fw)	0.24 ± 0.02	0.54 ± 0.01	<0.001 *
Proteins (g/100 g fw)	0.98 ± 0.01	0.40 ± 0.02	<0.001 *
Carbohydrates (g/100 g fw)	10.86 ± 0.31	13.89 ± 0.21	<0.001 *
Insoluble Dietary Fibers (g/100 g fw)	1.55 ± <0.01	2.37 ± 0.14	<0.001 *
Soluble Dietary Fibers (g/100 g fw)	0.11 ± <0.01	0.88 ± 0.06	<0.001 *
Total Dietary Fibers (g/100 g fw)	1.66 ± <0.01	3.26 ± 0.20	<0.001 *
Total Caloric Value (Kcal)	49.55 ± 1.15	62.02 ± 0.78	<0.001 *

Data represent means ± standard deviation from testing performed in triplicate. * *p* < 0.01; ** *p* < 0.05; fw: fresh weight.

**Table 2:** Sugar profile and content of edible parts from *Eugenia* fruits.

Sugar Content (mg/g fw)				
Class	Compound	*Eugenia calycina*	*Eugenia stigmatosa*	*p*-Value
Polyols	Mannitol	n.d.	n.d.	-
	Sorbitol	n.d.	n.d.	-
	Xylitol	n.d.	n.d.	-
Monosaccharides	Arabinose	n.d.	n.d.	-
	Fructose	37.83 ± 0.84	37.21 ± 0.97	0.454 ^ns^
	Glucose	38.76 ± 0.78	34.43 ± 0.82	0.003 *
	Rhamnose	0.47 ± 0.01	0.43 ± 0.01	0.006 *
Disaccharides	Maltose	n.d.	0.73 ± 0.06	-
	Sucrose	n.d.	n.d.	-
Maltooligosaccharides	Maltotriose	n.d.	t.r.	-
	Maltotetraose	n.d.	n.d.	-
	Maltopentaose	n.d.	n.d.	-
	Maltohexaose	n.d.	n.d.	-
	Maltoheptaose	n.d.	n.d.	-
Frutooligosaccharides	GF_2_	t.r.	n.d.	-
	GF_3_	t.r.	n.d.	-
	GF_4_	n.d.	n.d.	-
**Total**		**77.06 ± 1.64**	**72.07 ± 1.80**	**0.024**

Data represent means ± standard deviation from testing performed in triplicate. * *p* < 0.01; ns: not significant; GF2, GF3, and GF4 represent oligosaccharides composed of two, three, and four units of glucose (G) and fructose (F), respectively; fw: fresh weight; t.r.: traces (<limit of quantification); n.d.: not detected.

**Table 3:** Total content of bioactive compounds and antioxidant capacity in *Eugenia* fruits.

Parameters	*Eugenia calycina*	*Eugenia stigmatosa*	*p*-Value *
Total Phenolics (mg GAE/g fw)	3.95 ± 0.02	5.39 ± 0.07	<0.001 *
Total Flavonoids (mg CE/g fw)	2.82 ± 0.01	2.18 ± 0.07	<0.001 *
Monomeric Anthocyanins (mg C3G/g fw)	1.26 ± <0.01	0.01 ± <0.01	<0.001 *
Condensed Tannins (mg CE/g fw)	2.63 ± 0.33	4.36 ± 0.09	0.002 *
ORAC (µmol TE/g fw)	83.46 ± 3.11	44.00 ± 1.54	<0.001 *
FRAP (µmol TE/g fw)	53.29 ± 1.43	53.48 ± 0.93	0.857 ^ns^
ABTS (µmol TE/g fw)	40.66 ± 0.60	47.13 ± 1.60	0.002 *

Data represent means ± standard deviation from testing performed in triplicate. * *p* < 0.01; ns: not significant; fw: fresh weight; GAE: gallic acidic equivalent; CE: catechin equivalent; C3G: cyanidin-3-glucoside; TE: Trolox equivalent.

**Table 4:** Pearson correlation between bioactive compounds and antioxidant capacity of *Eugenia* fruits.

	TPC	TFC	MAC	CT	FRAP	ABTS
TFC	−0.981 *					
MAC	−0.998 *	0.989 *				
CT	0.964 *	−0.974 *	−0.965 *			
FRAP	0.075 ^ns^	−0.090 ^ns^	−0.096 ^ns^	−0.095 ^ns^		
ABTS	0.943 *	−0.988 *	−0.959 *	0.945 *	0.164 ^ns^	
ORAC	−0.992 *	0.984 *	0.995 *	−0.976 *	−0.023 ^ns^	−0.953 *

TPC: total phenolic content; TFC: total flavonoid content; MAC: monomeric anthocyanin; CT: condensed tannin; * *p* < 0.01 and ns: not significant.

**Table 5:** Phenolic compound profile and content in edible parts from *Eugenia* fruits.

Phenolic Compounds Content (µg/g fw)				
Class	Compound	*Eugenia calycina*	*Eugenia stigmatosa*	*p*-Value
Phenolic Acids	Caffeic acid	traces	n.d	-
	Ellagic acid	51.62 ± 1.29	n.d	-
	Gallic acid	n.d	4.42 ± 0.04	-
	Gentisic acid	1.90 ± 0.06	n.d	-
	Protocatechuic acid	2.70 ± 0.04	n.d	-
	Trans-cinnamic acid	n.d	0.20 ± 0.01	-
Flavonoids	Kaempferol	n.d	0.46 ± 0.01	-
	Quercetin	5.55 ± 0.07	0.75 ± 0.01	<0.001 *
	Quercetrin	n.d	1.53 ± 0.01	-
	Rutin	55.60 ± 1.05	10.45 ± 0.02	<0.001 *
Anthocyanins	Cyanidin-3-O-β-D-glucoside	242.97 ± 1.52	0.07 ± <0.01	<0.001 *
	Delphinidin-3-O-β-D-glucoside	traces	n.d	-
	Pelargonidin-3-O-β-D-glucoside	8.15 ± 0.10	n.d	-
**Total**		**368.49 ± 1.96**	**17.87 ± 0.06**	<0.001 *

Data represent means ± standard deviation from testing performed in triplicate. * *p* < 0.01; fw: fresh weight.

## Data Availability

The authors confirm that the data supporting the findings of this study are available within the article.

## References

[1] Brasil Ministério Do Meio Ambiente Flora Do Brasil. http://dspace.jbrj.gov.br.

[2] REFLORA Flora E Funga Do Brasil. https://floradobrasil.jbrj.gov.br/consulta/ficha.html?idDadosListaBrasil=10541.

[3] Miller V., Mente A., Dehghan M., Rangarajan S., Zhang X., Swaminathan S., Dagenais G., Gupta R., Mohan V., Lear S. (2017). Fruit, Vegetable, and Legume Intake, and Cardiovascular Disease and Deaths in 18 Countries (PURE): A Prospective Cohort Study. Lancet.

[4] de Araújo F.F., Neri-Numa I.A., de Paulo Farias D., da Cunha G.R.M.C., Pastore G.M. (2019). Wild Brazilian Species of *Eugenia* Genera (*Myrtaceae*) as an Innovation Hotspot for Food and Pharmacological Purposes. Food Res. Int..

[5] Zhang Q., Luna-Vital D., Gonzalez de Mejia E. (2019). Anthocyanins from Colored Maize Ameliorated the Inflammatory Paracrine Interplay between Macrophages and Adipocytes through Regulation of NF-ΚB and JNK-Dependent MAPK Pathways. J. Funct. Foods.

[6] Vendrame S., Klimis-Zacas D. (2015). Anti-Inflammatory Effect of Anthocyanins via Modulation of Nuclear Factor-ΚB and Mitogen-Activated Protein Kinase Signaling Cascades. Nutr. Rev..

[7] Santos-Buelga C., González-Paramás A.M., Oludemi T., Ayuda-Durán B., González-Manzano S. (2019). Plant Phenolics as Functional Food Ingredients. Adv. Food Nutr. Res..

[8] Fallah A.A., Sarmast E., Jafari T. (2020). Effect of Dietary Anthocyanins on Biomarkers of Oxidative Stress and Antioxidative Capacity: A Systematic Review and Meta-Analysis of Randomized Controlled Trials. J. Funct. Foods.

[9] Poulsen N.B., Lambert M.N.T., Jeppesen P.B. (2020). The Effect of Plant Derived Bioactive Compounds on Inflammation: A Systematic Review and Meta-Analysis. Mol. Nutr. Food Res..

[10] Baseggio A.M., Kido L.A., Viganó J., Carneiro M.J., Lamas C.d.A., Martínez J., Sawaya A.C.H.F., Cagnon V.H.A., Maróstica Júnior M.R. (2022). Systemic Antioxidant and Anti-Inflammatory Effects of Yellow Passion Fruit Bagasse Extract during Prostate Cancer Progression. J. Food Biochem..

[11] Romero-Díez R., Matos M., Rodrigues L., Bronze M.R., Rodríguez-Rojo S., Cocero M.J., Matias A.A. (2019). Microwave and Ultrasound Pre-Treatments to Enhance Anthocyanins Extraction from Different Wine Lees. Food Chem..

[12] Arruda H.S., Silva E.K., Pereira G.A., Angolini C.F.F., Eberlin M.N., Meireles M.A.A., Pastore G.M. (2019). Effects of High-Intensity Ultrasound Process Parameters on the Phenolic Compounds Recovery from Araticum Peel. Ultrason. Sonochem..

[13] UN General Assembly Transforming Our World: The 2030 Agenda for Sustainable Development. https://www.refworld.org/docid/57b6e3e44.html.

[14] Mudgil D., Barak S. (2019). Classification, Technological Properties, and Sustainable Sources. Dietary Fiber: Properties, Recovery, and Applications.

[15] World Health Organization (2023). Carbohydrate Intake for Adults and Children: WHO Guideline.

[16] Food and Drug Administration (FDA) Labeling & Nutrition—Guidance for Industry: A Food Labeling Guide (14. Appendix F: Calculate the Percent Daily Value for the Appropriate Nutrients). Center for Food Safety and Applied Nutrition. www.fda.gov/FoodLabelingGuide.

[17] Loubet Filho P.S., Baseggio A.M., Vuolo M.M., Reguengo L.M., Telles Biasoto A.C., Correa L.C., Junior S.B., Alves Cagnon V.H., Betim Cazarin C.B., Maróstica Júnior M.R. (2022). Gut Microbiota Modulation by Jabuticaba Peel and Its Effect on Glucose Metabolism via Inflammatory Signaling. Curr. Res. Food Sci..

[18] Bagetti M., Facco E.M.P., Piccolo J., Hirsch G.E., Rodriguez-Amaya D., Kobori C.N., Vizzotto M., Emanuelli T. (2011). Physicochemical Characterization and Antioxidant Capacity of Pitanga Fruits (*Eugenia uniflora* L.). Food Sci. Technol..

[19] Baliga M.S., Bhat H.P., Baliga B.R.V., Wilson R., Palatty P.L. (2011). Phytochemistry, Traditional Uses and Pharmacology of *Eugenia jambolana* Lam. (Black Plum): A Review. Food Res. Int..

[20] Do Nascimento V.T., De Moura N.P., Da Silva Vasconcelos M.A., Maciel M.I.S., De Albuquerque U.P. (2011). Chemical Characterization of Native Wild Plants of Dry Seasonal Forests of the Semi-Arid Region of Northeastern Brazil. Food Res. Int..

[21] Muir J.G., Rose R., Rosella O., Liels K., Barrett J.S., Shepherd S.J., Gibson P.R. (2009). Measurement of Short-Chain Carbohydrates in Common Australian Vegetables and Fruits by High-Performance Liquid Chromatography (HPLC). J. Agric. Food Chem..

[22] Laughlin M.R. (2014). Normal Roles for Dietary Fructose in Carbohydrate Metabolism. Nutrients.

[23] Biesiekierski J.R., Rosella O., Rose R., Liels K., Barrett J.S., Shepherd S.J., Gibson P.R., Muir J.G. (2011). Quantification of Fructans, Galacto-Oligosacharides and Other Short-Chain Carbohydrates in Processed Grains and Cereals. J. Hum. Nutr. Diet..

[24] Zhao D., Li S., Han X., Li C., Ni Y., Hao J. (2020). Physico-Chemical Properties and Free Amino Acids Profiles of Six Wolfberry Cultivars in Zhongning. J. Food Compos. Anal..

[25] Nehring P., Siluana K.T.S., Schulz M., Della Betta F., Gonzaga L.V., Vitali L., da Silva M., Micke G.A., Costa A.C.O., Fett R. (2022). Grumixama (*Eugenia brasiliensis* Lamarck) Functional Phytochemicals: Effect of Environmental Conditions and Ripening Process. Food Res. Int..

[26] Vidović B.B., Marčetić M.D., Djuriš J., Milinčić D.D., Kostić A., Pešić M.B. (2023). Goji Berries: Valuable Sources of Nutrients and Bioactive Compounds. Sustain. Food Sci. A Compr. Approach.

[27] Vona R., Pallotta L., Cappelletti M., Severi C., Matarrese P. (2021). The Impact of Oxidative Stress in Human Pathology: Focus on Gastrointestinal Disorders. Antioxidants.

[28] Ramos A.S., Mar J.M., da Silva L.S., Acho L.D.R., Silva B.J.P., Lima E.S., Campelo P.H., Sanches E.A., Bezerra J.A., Chaves F.C.M. (2019). Pedra-Ume Caá Fruit: An Amazon Cherry Rich in Phenolic Compounds with Antiglycant and Antioxidant Properties. Food Res. Int..

[29] Braga E.C.d.O., Pacheco S., de Araujo Santiago M.C.P., de Oliveira Godoy R.L., de Jesus M.S.C., de Carvalho Martins V., da Costa Souza M., Porte A., Borguini R.G. (2023). Bioactive Compounds of *Eugenia punicifolia* Fruits: A Rich Source of Lycopene. Braz. J. Food Technol..

[30] Araujo N.M.P., Silva E.K., Arruda H.S., Rodrigues de Morais D., Angela A., Meireles M., Pereira G.A., Pastore G.M. (2021). Recovering Phenolic Compounds from *Eugenia calycina* Cambess Employing High-Intensity Ultrasound Treatments: A Comparison among Its Leaves, Fruit Pulp, and Seed as Promising Sources of Bioactive Compounds. Sep. Purif. Technol..

[31] Araujo N.M.P., Arruda H.S., dos Santos F.N., de Morais D.R., Pereira G.A., Pastore G.M. (2020). LC-MS/MS Screening and Identification of Bioactive Compounds in Leaves, Pulp and Seed from *Eugenia calycina* Cambess. Food Res. Int..

[32] Lu Y., Guo S., Zhang F., Yan H., Qian D.-W., Shang E.-X., Wang H.-Q., Duan J.-A. (2021). Nutritional Components Characterization of Goji Berries from Different Regions in China. J. Pharm. Biomed. Anal..

[33] Popoola O.O. (2022). Phenolic Compounds Composition and in Vitro Antioxidant Activity of Nigerian *Amaranthus Viridis* Seed as Affected by Autoclaving and Germination. Meas. Food.

[34] Bonin A.M.F., Ávila S., Etgeton S.A.P., de Lima J.J., dos Santos M.P., Grassi M.T., Krüger C.C.H. (2024). Ripening Stage Impacts Nutritional Components, Antiglycemic Potential, Digestibility and Antioxidant Properties of Grumixama (*Eugenia brasiliensis* Lam.) Fruit. Food Res. Int..

[35] Chemat F., Rombaut N., Sicaire A.G., Meullemiestre A., Fabiano-Tixier A.S., Abert-Vian M. (2017). Ultrasound Assisted Extraction of Food and Natural Products. Mechanisms, Techniques, Combinations, Protocols and Applications. A Review. Ultrason. Sonochem..

[36] Pinela J., Prieto M.A., Pereira E., Jabeur I., Barreiro M.F., Barros L., Ferreira I.C.F.R. (2019). Optimization of Heat- and Ultrasound-Assisted Extraction of Anthocyanins from Hibiscus Sabdariffa Calyces for Natural Food Colorants. Food Chem..

[37] Albuquerque B.R., Pinela J., Pereira C., Mandim F., Heleno S., Oliveira M.B.P.P., Barros L. (2024). Recovery of Anthocyanins from *Eugenia* spp. Fruit Peels: A Comparison between Heat- and Ultrasound-Assisted Extraction. Sustain. Food Technol..

[38] Ma Y., Feng Y., Diao T., Zeng W., Zuo Y. (2020). Experimental and Theoretical Study on Antioxidant Activity of the Four Anthocyanins. J. Mol. Struct..

[39] Schmidt H.d.O., Rockett F.C., Klen A.V.B., Schmidt L., Rodrigues E., Tischer B., Augusti P.R., de Oliveira V.R., da Silva V.L., Flôres S.H. (2020). New Insights into the Phenolic Compounds and Antioxidant Capacity of Feijoa and Cherry Fruits Cultivated in Brazil. Food Res. Int..

[40] Lang Y., Gao N., Zang Z., Meng X., Lin Y., Yang S., Yang Y., Jin Z., Li B. (2024). Classification and Antioxidant Assays of Polyphenols: A Review. J. Future Foods.

[41] Ayoub M., De Camargo A.C., Shahidi F. (2016). Antioxidants and Bioactivities of Free, Esterified and Insoluble-Bound Phenolics from Berry Seed Meals. Food Chem..

[42] Toshima S., Hirano T., Kunitake H. (2021). Comparison of Anthocyanins, Polyphenols, and Antioxidant Capacities among Raspberry, Blackberry, and Japanese Wild *Rubus* Species. Sci. Hortic..

[43] Frutos M.J., Rincón-Frutos L., Valero-Cases E. (2019). Rutin. Nonvitamin and Nonmineral Nutritional Supplements.

[44] Kang I., Buckner T., Shay N.F., Gu L., Chung S. (2016). Improvements in Metabolic Health with Consumption of Ellagic Acid and Subsequent Conversion into Urolithins: Evidence and Mechanisms. Adv. Nutr..

[45] Machado A.P.D.F., Pereira A.L.D., Barbero G.F., Martínez J. (2017). Recovery of Anthocyanins from Residues of *Rubus Fruticosus, Vaccinium myrtillus* and *Eugenia brasiliensis* by Ultrasound Assisted Extraction, Pressurized Liquid Extraction and Their Combination. Food Chem..

[46] Flores G., Dastmalchi K., Paulino S., Whalen K., Dabo A.J., Reynertson K.A., Foronjy R.F., D’Armiento J.M., Kennelly E.J. (2012). Anthocyanins from *Eugenia brasiliensis* Edible Fruits as Potential Therapeutics for COPD Treatment. Food Chem..

[47] Dametto A.C., Agustoni D., Moreira T.F., Plaza C.V., Prieto A.M., Silva T.G.A., Souza F.O., Boralle N., Maria Sorbo J., Silva D.H.S. (2017). Chemical Composition and in Vitro Chemoprevention Assessment of *Eugenia jambolana* Lam. (*Myrtaceae*) Fruits and Leaves. J. Funct. Foods.

[48] Teixeira L.D.L., Bertoldi F.C., Lajolo F.M., Hassimotto N.M.A. (2015). Identification of Ellagitannins and Flavonoids from *Eugenia brasilienses* Lam. (Grumixama) by HPLC-ESI-MS/MS. J. Agric. Food Chem..

[49] Hariri M., Amirkalali B., Gholami A. (2024). Effects of Purified Anthocyanins Supplementation on Serum Concentration of Inflammatory Mediators: A Systematic Review and Dose-Response Meta-Analysis on Randomized Clinical Trials. Phytother. Res..

[50] Mohammadi N., Farrell M., O’Sullivan L., Langan A., Franchin M., Azevedo L., Granato D. (2024). Effectiveness of Anthocyanin-Containing Foods and Nutraceuticals in Mitigating Oxidative Stress, Inflammation, and Cardiovascular Health-Related Biomarkers: A Systematic Review of Animal and Human Interventions. Food Funct..

[51] World Health Organization (2004). Fruit and Vegetables for Health: Report of a Joint FAO/WHO Workshop.

[52] Paludo M.C., Colombo R.C., Filho J.T., Hermosín-Gutiérrez I., Ballus C.A., Godoy H.T. (2019). Optimizing the Extraction of Anthocyanins from the Skin and Phenolic Compounds from the Seed of Jabuticaba Fruits (*Myrciaria jabuticaba* (Vell.) O. Berg) with Ternary Mixture Experimental Designs. J. Braz. Chem. Soc..

[53] Instituto Adolfo Lutz (IAL) (2008). Métodos Físico-Químicos Para Análise de Alimentos.

[54] Bligh E.G., Dyer W.J. (1959). A Rapid Method of Total Lipid Extraction and Purification. Can. J. Biochem. Physiol..

[55] AACC (2010). American Association of Cereal Chemist International AACC Approved Methods of Analysis.

[56] Merril A.L., Watt B.K. (1973). Energy Value of Foods: Basis and Derivation.

[57] Pereira G.A., Arruda H.S., de Morais D.R., Eberlin M.N., Pastore G.M. (2018). Carbohydrates, Volatile and Phenolic Compounds Composition, and Antioxidant Activity of Calabura (*Muntingia calabura* L.) Fruit. Food Res. Int..

[58] Singleton V.L., Rossi J.A. (1965). Colorimetry of Total Phenolics with Phosphomolybdic-Phosphotungstic Acid Reagents. Am. J. Enol. Vitic..

[59] Vasco C., Ruales J., Kamal-Eldin A. (2008). Total Phenolic Compounds and Antioxidant Capacities of Major Fruits from Ecuador. Food Chem..

[60] Zhishen J., Mengcheng T., Jianming W. (1999). The Determination of Flavonoid Contents in Mulberry and Their Scavenging Effects on Superoxide Radicals. Food Chem..

[61] Giusti M.M., Wrolstad R.E. (2001). Characterization and Measurement of Anthocyanins by UV-Visible Spectroscopy. Current Protocols in Food Analytical Chemistry.

[62] Arruda H.S., Pereira G.A., de Morais D.R., Eberlin M.N., Pastore G.M. (2018). Determination of Free, Esterified, Glycosylated and Insoluble-Bound Phenolics Composition in the Edible Part of Araticum Fruit (*Annona crassiflora* Mart.) and Its by-Products by HPLC-ESI-MS/MS. Food Chem..

[63] Guerra-Ramírez D., González-García K.E., Medrano-Hernández J.M., Famiani F., Cruz-Castillo J.G. (2021). Antioxidants in Processed Fruit, Essential Oil, and Seed Oils of Feijoa. Not. Bot. Horti Agrobot. Cluj-Napoca.

[64] Re R., Pellegrini N., Proteggente A., Pannala A., Yang M., Rice-Evans C. (1999). Antioxidant Activity Applying an Improved ABTS Radical Cation Decolorization Assay. Free Radic. Biol. Med..

[65] Dávalos A., Gómez-Cordovés C., Bartolomé B. (2004). Extending Applicability of the Oxygen Radical Absorbance Capacity (ORAC-Fluorescein) Assay. J. Agric. Food Chem..

